# Resting Motor Threshold, MEP and TEP Variability During Daytime

**DOI:** 10.1007/s10548-018-0662-7

**Published:** 2018-07-17

**Authors:** Esther M. ter Braack, Annika A. de Goede, Michel J. A. M. van Putten

**Affiliations:** 1 Department of Clinical Neurophysiology, Technical Medical Centre, University of Twente, Carré CR 3.623, P.O. 217, 7500 AE Enschede, The Netherlands; 2 Department of Clinical Neurophysiology, Medisch Spectrum Twente, Enschede, The Netherlands

**Keywords:** TMS, EEG, TMS evoked potential, Daytime variation

## Abstract

Humans show a variation in physiological processes during the day. To reliably assess (changes in) cortical excitability with transcranial magnetic stimulation (TMS), it is relevant to know the natural variation in TMS readouts during the day. In case of significant daytime variations, this should be taken into account when scheduling (follow-up) measurements. This study aims to evaluate the influence of the time of day on the resting motor threshold (RMT), motor evoked potential (MEP) and TMS evoked potential (TEP) in healthy controls. TMS–EMG–EEG was recorded in 16 healthy subjects. At both motor cortices, we administered 75 pulses at an intensity of 110% RMT. Subjects were stimulated during five sessions in one day (8:00 AM, 10:30 AM, 1:00 PM, 3:30 PM and 6:00 PM) while keeping the stimulation intensity constant. We compared the TEP waveforms between the five sessions with a cluster-based permutation analysis, and the RMT and MEP amplitude with rmANOVA. In general there were no significant differences between the five sessions in the RMT, MEP amplitude or TEP. Only for the left side, N100 amplitude was larger at 3:30 PM than 10:30 AM. The standard deviation of the P30 and N100 amplitude was significantly higher between subjects within one session than within single subjects during the day. The TEP is highly reproducible during the day, with a low intra-individual variation compared to the inter-individual variation. In addition, we found no significant variation of the RMT and MEP amplitude between multiple sessions on one day.

## Introduction

Humans, and animals and plants as well, show a variation in physiological processes during the day. This circadian rhythm is regulated by our biological clock, resulting in diurnal fluctuations in for instance hormone secretion, blood pressure, but also alertness (http://www.Nobelprize.org). Some epilepsies show a relationship with sleep stages or the transition between sleep and wakefulness, of which juvenile myoclonic epilepsy (JME) is the most typical example where seizures occur predominantly after awakening in the morning. Besides the interaction between sleep and epilepsy, the time of day is also correlated with seizure occurrence in some focal epilepsy types (Hofstra and de Weerd 2009; Mirzoev et al. 2012; van Campen et al. 2015). This diurnal distribution of seizures is mainly evident for temporal lobe epilepsy, with a peak in seizure occurrence in the late afternoon (Durazzo et al. 2008; Hofstra et al. 2009; Pavlova et al. 2004).

Epilepsy can be characterized as a disease resulting from an imbalance between cortical excitation and inhibition. Transcranial magnetic stimulation (TMS) is a well-established technique to non-invasively activate brain areas (Barker et al. 1985), and is a promising method to assess cortical excitability, which we here define as the strength of the response of cortical neurons to an external input. The resting motor threshold (RMT) or the MEP amplitude following a neuromodulatory paradigm (paired-pulse TMS) can be used as readouts of cortical excitability. Combining TMS with EEG has also become available (Ilmoniemi and Kičić 2010; Miniussi and Thut 2010), opening novel possibilities to study cortical excitability. A single TMS pulse induces a response in the EEG, and after averaging over multiple pulses, the TMS evoked potential (TEP) is obtained. TMS–EEG could provide a more direct measure of cortical excitability than measuring the MEP (Bonato et al. 2006; Ferreri et al. 2011; Ilmoniemi and Kičić 2010), as it is not influenced by the excitability of corticospinal and spinal neurons. The TEP has been shown to change after administration of GABA-ergic drugs as well as anti-epileptic drugs (Premoli et al. 2017, 2014).

TMS is considered to be a candidate tool for a new biomarker in epilepsy (Bauer et al. 2014; Chen et al. 2008; Engel 2008; Kimiskidis 2016; Manganotti and del Felice 2013). To reliably assess (changes in) cortical excitability, it is relevant to know the natural variation in TMS readouts during the day. When there is a significant effect of time-of-day, scheduling follow-up measurements should be performed at approximately the same time in the morning or afternoon. Previous studies have shown that the RMT and MEP remains constant during the day (Doeltgen and Ridding 2010; Koski et al. 2005; Lang et al. 2011; Strutton et al. 2003). It is known that the TEP changes when different brain areas are stimulated, and also when the stimulation intensity or stimulation angle is varied (Casarotto et al. 2010). While the TEP after stimulating the motor cortex shows high repeatability comparing the first and last part of a TMS–EEG session (Casarotto et al. 2010; Kerwin et al. 2018) and is highly reproducible after 1 week when stimulating parameters are kept constant (Casarotto et al. 2010; Lioumis et al. 2009), diurnal variations of the TEP have not been systematically studied.

The variation of one component of the TEP during daytime has been studied in healthy subjects as a part of larger experimental protocols focusing on the effects of sleep deprivation. The P30 of the TEP did not differ between 9 AM and 3 PM, but was significantly higher at 9 PM compared to 9 AM in five out of six subjects (Huber et al. 2012). In a subsequent study by the same research group, the amplitude and slope of the P30 was significantly lower at 9 PM compared to 5 PM, but no difference was found between 11 AM and 5 PM (Ly et al. 2016).

The variation of the TEP using multiple measurements during daytime has not been reported before. In this study we investigated the daytime variation of the RMT, MEP amplitude and the TEP after motor cortex stimulation by measuring these responses at 2.5 h intervals between 8 AM and 6 PM in a group of healthy volunteers.

## Materials and Methods

The experimental protocol was approved by the local ethics committee (Medisch Spectrum Twente, Enschede, the Netherlands) and was in accordance with the declaration of Helsinki and the guidelines for the use of TMS in clinical practice and research (Rossi et al. 2009). All subjects gave written informed consent.

### Subjects

Nineteen healthy subjects participated in this study. One of these 19 subjects had a syncope at the start of the first TMS session and was excluded from the study. The remaining 18 subjects tolerated the TMS protocol well. Part of the data from these 18 subjects was previously presented, for a different objective (ter Braack et al. 2013, 2016). Another two subjects were excluded from the analysis, since one subject had a missing session for both targets, and one subject had two missing sessions for one target, both due to technical problems with the navigation system. Sixteen subjects (11 males, mean age 28 years, all right-handed) were therefore included in the analysis.

### Experimental Protocol

Subjects were seated in a chair, with their hands pronated in a relaxed position. They kept their eyes open, focusing on a marked point on the wall. Subjects were asked to refrain from alcohol 2 days and from caffeinated drinks 12 h prior to measurements. Subjects were only measured if they had a good night of sleep. Each subject underwent 1 day of measurements, divided in 5 sessions. The sessions took place at 8:00 AM, 10:30 AM, 1:00 PM, 3:30 PM and 6:00 PM. In three subjects we repeated the first session a week later to confirm previous findings of a good reproducibility (Lioumis et al. 2009).

### Stimulation

Single biphasic TMS pulses, with pulse duration of 400 µs and inter-pulse interval of ~ 4 s, were delivered manually using a 70 mm figure-of-eight air film coil and a Magstim Rapid^2^ stimulator (The Magstim Company Ltd, Whitland, United Kingdom). The maximum stimulator output was 0.8 T. The coil was placed tangentially over the hot-spot of the abductor digiti minimi muscle (ADM) in the left and right hemisphere. The two targets are referred to as motor cortex left (MCL) and motor cortex right (MCR). The handle was pointing backwards and laterally at a 45° angle away from the midline. At both targets we applied 75 TMS pulses at a stimulation intensity of 110% of the RMT of the ADM hotspot. This stimulation intensity was kept constant during the day. The motor threshold was defined as the lowest stimulus intensity that produced at least five MEPs of at least 50 µV out of 10 consecutive stimuli (Rossini et al. 1994). In four of the 16 subjects, a TMS intensity of 110% RMT could not be given due to a too high threshold in session 1. In those subjects, the TMS intensity during the protocol was set to the maximum output of the stimulator (0.8 T), corresponding to 100–108% RMT. During TMS–EEG, all subjects wore protective earplugs, and noise created from the coil click was played through headphones at 95 dB to mask the sound of the TMS pulses (ter Braack et al. 2015). In addition, a thin layer of foam was placed between the coil and head of the subject to minimize bone conduction.

### TMS Targeting

Positioning of the coil was achieved using a robot-navigated system (Smartmove, ANT Neuro, Enschede, Netherlands), with an accuracy of 1 mm in every direction. A headband carrying four passive reflective markers was fixed to the head of the subject and tracked by a Polaris infrared camera system (Northern Digital Inc., Waterloo, Ontario, Canada). The robot and the tracking system were registered to a common coordinate system using a calibration procedure. The robot-guided TMS coil was added to the coordinate system by registration of three reference positions on the coil using a tracking pointer. In all subjects, a 1.5 T MRI scan of the head was available. The MRI scan was used to create a subject-specific head model; this model was then registered to the subject’s head and the coordinate system by collecting three landmarks and 300 additional points on the scalp with a tracking pointer.

### EEG and EMG Recording During TMS

The EEG was recorded continuously during TMS using a full-band amplifier (TMSi, Oldenzaal, Netherlands) and a TMS-compatible 64-electrode cap (ANT Neuro, Enschede, Netherlands). The EEG cap stayed in place during the whole day. Impedances were kept below 5 kΩ. The ground electrode was placed between electrode positions Fz and Fpz. We used a common average reference for the recordings. In our data, a single TMS pulse produced a magnetic stimulation artifact of 1–2 mV, lasting approximately 3 ms using the full-band amplifier. To determine the hotspot and RMT, surface electrodes were placed in a belly–tendon montage over the right and left ADM muscle. The ground electrode was placed on the upper side of the wrist. We recorded the EMG using an additional amplifier (TMSi, Oldenzaal, Netherlands) connected to the EEG amplifier, ensuring synchronized measurements. The EEG and EMG signals were low-pass filtered with an anti-aliasing filter with a cut-off frequency of 550 Hz and sampled at 2048 Hz.

### Evoked Potential Analysis

EMG and EEG analysis was performed using Matlab (The Mathworks, Natick, MA, USA). To analyze the MEP, peak-to-peak amplitudes were calculated and averaged per session. Trials containing muscle pre-activation, defined as EMG activity larger than 50 µV in the 50 ms preceding a single pulse, were excluded.

TMS evoked potentials were analyzed using the common average reference. Trials were defined from 50 ms before to 300 ms after every TMS pulse, resulting in 75 trials for both targets and each session. We applied single-trial principal component analysis (PCA) to remove the first large TMS artifact, caused by the magnetic pulse, and the second TMS artifact, believed to be caused by muscle activation on the scalp. A detailed description of this PCA method can be found in a previous study (ter Braack et al. 2013). In short, we performed PCA using 40 calculated components on each individual trial, with the first component having the largest variance and the 40th component having the lowest variance. We then removed the first four components, containing the large amplitude artifacts, from the trial to obtain a signal which is almost artifact-free. After PCA, the trials were filtered with a fourth order Butterworth bandpass filter between 1 and 45 Hz and averaged per session.

To investigate the variation of the P30 and N100 amplitude during the day, we determined the standard deviation at the latency of the maximum amplitude of both components at electrode Cz. The standard deviation was determined for the response on group level for each session and then averaged over sessions, resulting in an average inter-individual variation of the P30 and N100 amplitude during the day. We also determined this standard deviation for each individual subject for the average response over all five sessions and then averaged over subjects, resulting in an average intra-individual variation of the P30 and N100 amplitude during the day.

### Statistical Analysis

For all statistical analyses a *p*-value below 0.05 was considered statistically significant, except when a correction for multiple comparisons was applied.

RMT and mean MEP amplitude between the five sessions was compared using one-way repeated measures ANOVA with Greenhouse–Geisser correction for both left and right motor cortex stimulation. Two-way repeated measures ANOVA was used to test for differences between both hemispheres. Since RMT was occasionally above the maximum output of the stimulator, and statistical analysis of bounded variables is challenging, those subjects were excluded for RMT statistics. A total of 13 and 15 subjects were included in the RMT analysis for the left and right hemispheres, respectively.

We compared the standard deviation of the P30 and N100 on group level (five sessions) with the standard deviation of the P30 and N100 on single subject level during the day (16 subjects) using an independent *t*-test.

All subjects were included in the TEP statistics. To compare the reproducibility of the total TEP waveform (0–300 ms) between the five sessions, we used multiple dependent *t*-tests at the electrode level. A cluster-based permutation analysis (Maris and Oostenveld 2007) was applied, as implemented in FieldTrip (http://fieldtrip.fcdonders.nl/), which enables analysis of the whole waveform on all electrodes. In short, a dependent *t*-test comparing the TEPs from the five sessions was performed for each time sample and each EEG electrode. Only *t*-values with a clustering *p*-value < 0.05 were considered for clustering. Clustering of *t*-values was based on adjacent time bins and neighboring electrodes. Within each cluster, the *t*-values of the included electrodes were summed, and this sum was used for statistical comparison. A permutation test was performed, randomly assigning the TEPs from the 16 subjects to two different groups (for example session 1 and session 2 are now randomly shuffled) and repeating statistical testing for 1500 times. These permutation results are then combined to form a distribution of summed clusters *t*-values. Clusters in the original data set were considered to show a non-significant trend if < 5% of the permutations in the distribution had a cluster-level statistic larger than the statistic in the original data set, i.e. with an alpha *p*-value of < 0.05. Only clusters with a *p*-value < 0.005 were only considered statistically significant, as *p*-values were afterwards Bonferroni corrected for 10 comparisons (all sessions were compared to each other). The same procedure was repeated for the time-intervals 20–35 and 80–140 ms to evaluate the P30 and N100 components of the TEP in further detail.

## Results

**Fig. 1:**
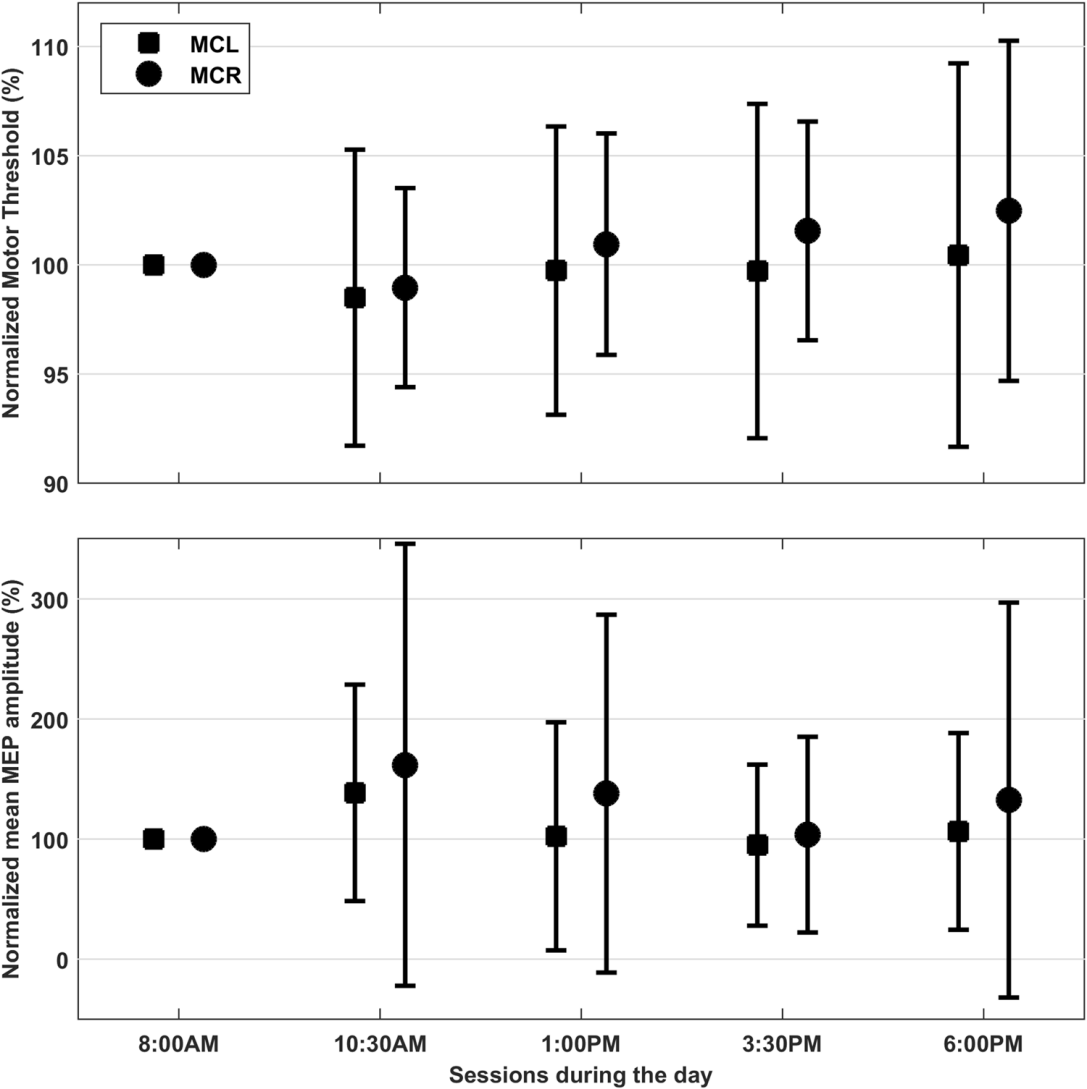
RMT and MEP variation during the day. The normalized RMT (top) and the normalized mean MEP amplitude (bottom) for the left (squares) and right (circles) hemisphere for all five sessions. The RMT and MEP amplitude were normalized with respect to the first session for every subject and then averaged over subjects. For RMT we excluded subjects with an RMT higher than 100% maximum stimulator output during one or more sessions, resulting in 13 subjects for the left hemisphere and 15 subjects for the right hemisphere. Error bars indicate the standard deviation

The mean RMT for the first session was 79% for the left hemisphere and 78% for the right hemisphere (Table 1). The RMT normalized with respect to the first session is presented in Fig. 1. The RMT showed no differences between the left and right hemisphere (*F*(1.91,22.86) = 0.09, *p* = 0.90) and did not change significantly during the day (left hemisphere: *F*(1.97,23.66) = 0.41, *p* = 0.67; right hemisphere: *F*(2.08,29.11) = 1.99, *p* = 0.15). The MEP amplitude showed no differences between the left and right hemisphere (*F*(2.26,33.82) = 0.16, *p* = 0.88) and did not change significantly during the day (left hemisphere: *F*(1.94,29.11) = 1.50, *p* = 0.24; right hemisphere: *F*(2.34,35.18) = 1.94, *p* = 0.15). In all subjects MEPs were evoked continuously, except in one where MEPs were absent (amplitude < 50 µV) for left hemisphere stimulation at 3:30 PM and 6:00 PM. The mean MEP amplitude normalized with respect to the first session is presented in Fig. 1.

**Table 1:** Resting motor thresholds and used TMS intensity (left/right) for all subjects

Subject	Session 1	Session 2	Session 3	Session 4	Session 5	TMS intensity
1	87/78	89/77	92/77	95/76	93/73	96/86
2	80 /65	76/68	86/67	85/68	84/68	88/71
3	70/67	72/67	72/68	71/72	72/72	77/74
4	70/82	72/81	72/80	73/78	73/83	77/91
5	86/87	79/88	75/93	75/93	76/100	95/96
6	65/61	58/60	62/61	59/60	58/57	72/68
7^a^	97^a^/97^a^	95/99	93/100	95/99	96/100	100/100
8	80/84	84/83	80/81	79/86	78/86	88/93
9^a,b^	94^a^/75	100/75	> 100/85	> 100/82	> 100/90	100/83
10	88/91	89/85	85/85	87/82	89/83	97/100
11^a,b,c^	93^a^/> 100^a^	96/100	> 100/> 100	94/> 100	95/88	100/100
12	74/70	75/73	77/71	78/71	83/70	82/77
13	77/76	64/65	68/71	66/76	64/73	85/85
14	84/73	86/73	88/74	86/76	92/74	93/81
15	74/75	78/74	78/78	80/78	79/80	82/83
16^a,b^	98^a^/93^a^	> 100/93	100/93	100/93	> 100/94	100/100

Resting motor threshold and applied TMS intensity during the protocol in % of maximal stimulator output (0.8 T) for left/right hemisphere.

^a^Protocol intensity of 110% RMT not possible

^b^Subject not included in RMT analysis left hemisphere

^c^Subject not included in RMT analysis right hemisphere

**Fig. 2:**
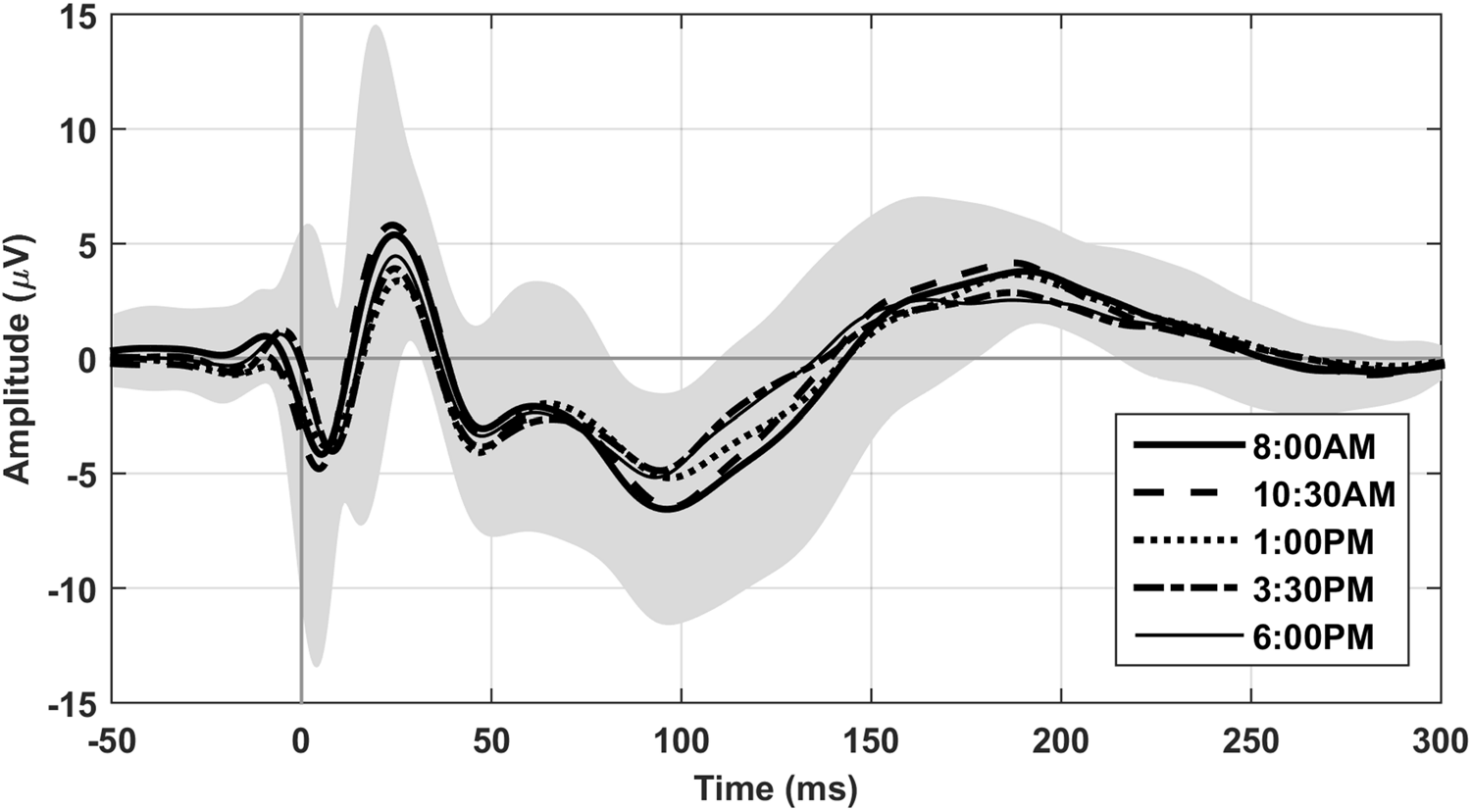
TEP on group level during the day. The TEP at electrode Cz on a group level during five sessions on 1 day after stimulating the left motor cortex. The TEP looks very similar during all five sessions, showing the typical components N15, P30, N45, P60, N100 and P180. The grey area represents the standard deviation on group level for the first session at 8:00 AM

**Fig. 3:**
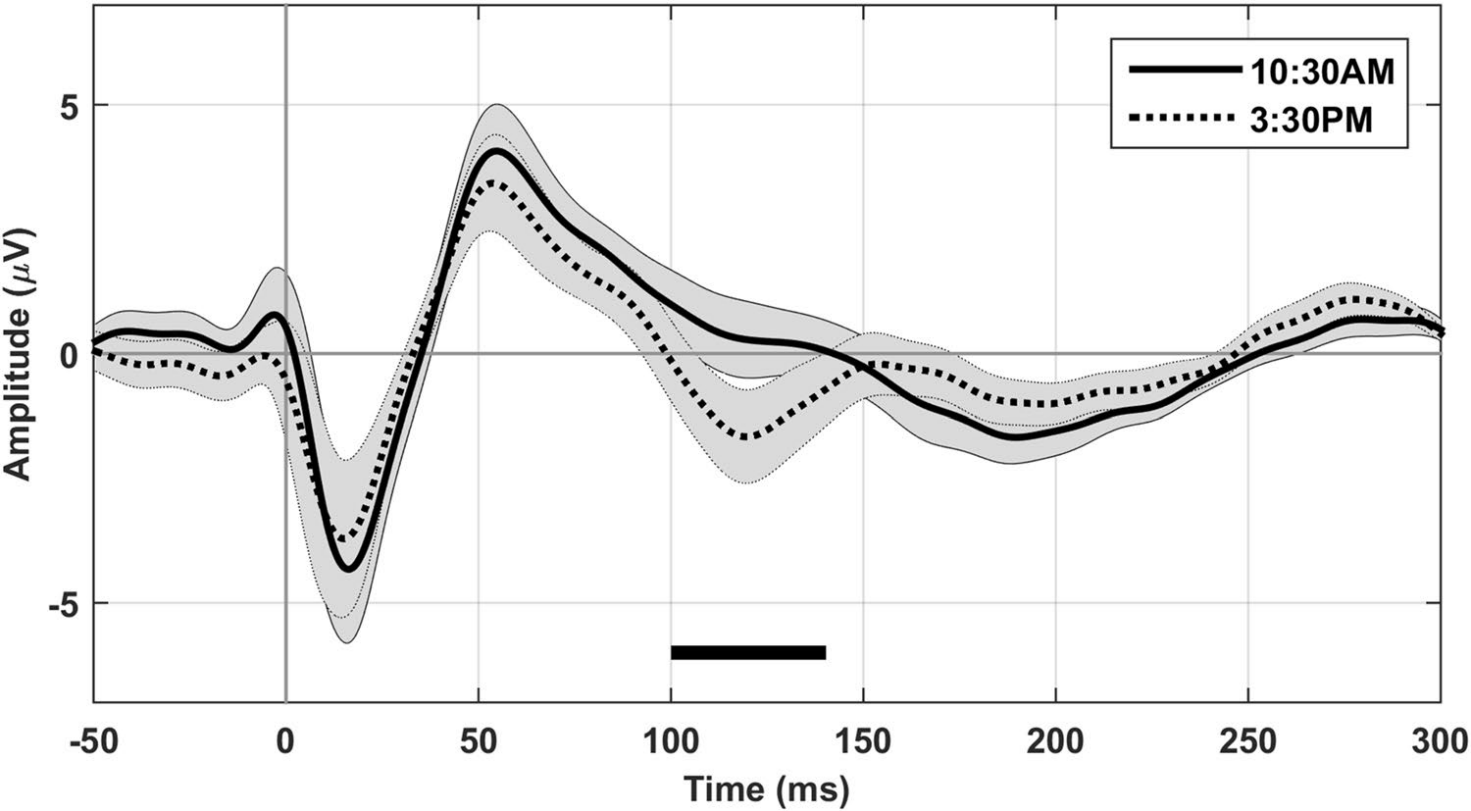
Difference in N100 between session 2 and 4. The TEP on group level in session 2 (10:30 AM, solid line) and session 4 (3:30 PM, dotted line) at electrode PO3 after stimulating the left motor cortex. The grey area represents the standard error. PO3 is the electrode with the largest difference in amplitude within the significant cluster, of which the duration is indicated by the black bar at the N100 component

**Fig. 4:**
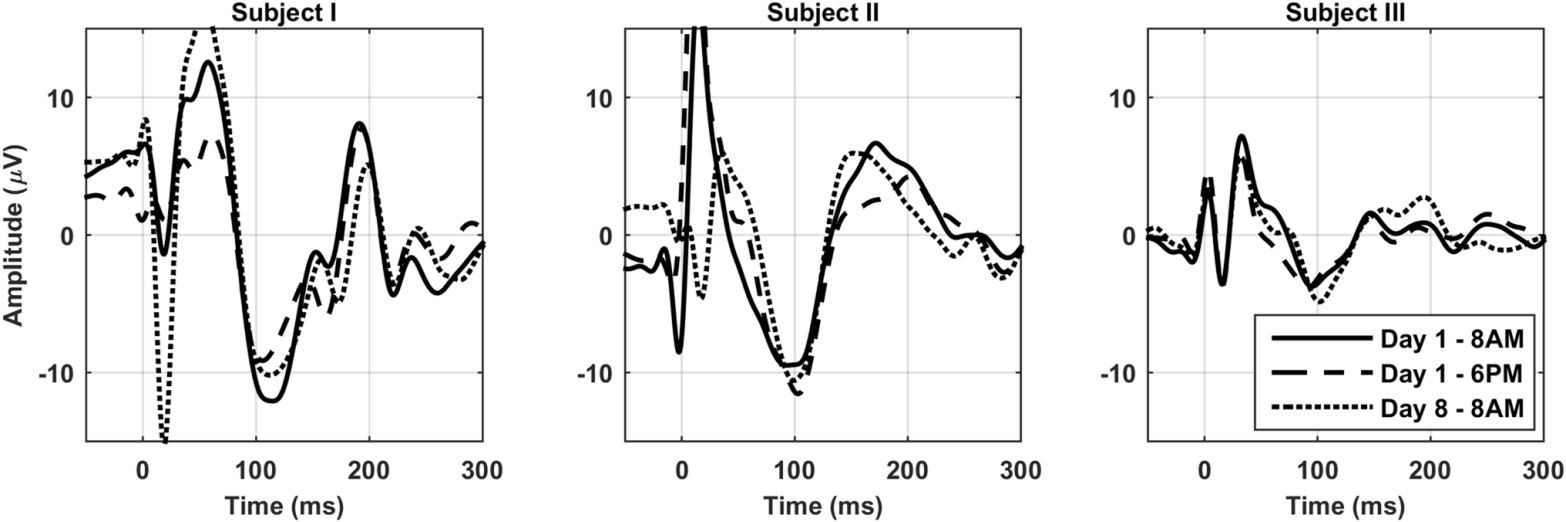
TEP after 1 week. The TEP at electrode Cz in 3 subjects after stimulating the right motor cortex at 8 AM and 6 PM on day 1, and at 8 AM after 1 week (day 8)

Figure 2 shows the TEP for all five sessions at electrode Cz averaged over subjects after stimulating the left motor cortex. The response was very constant during the day, showing a similar waveform in all five sessions in each subject. We found no significant differences in the TEP between the five sessions for MCR stimulation on a group level. Also for MCL stimulation, the majority of sessions showed no significant differences, except for 10:30 AM compared to 3:30 PM (*p* = 0.0046). One significant positive cluster was found left occipitally around 110–120 ms, corresponding to a larger (more negative) amplitude of the N100 component at 3:30 PM. When focusing on the N100, the same positive cluster was found to be significant (10:30 AM compared to 3:30 PM for MCL, *p* = 0.001), located left occipitally. The electrode with the largest amplitude difference within the cluster (PO3) is shown in Fig. 3. When focusing on the P30, no additional significant clusters were found. In addition we found some non-significant trends for the N100 8:00 AM versus 3:30 PM for MCL: positive cluster left occipitally (interval 0–300 ms (*p* = 0.02); interval 80–140 ms (*p* = 0.01)), and 8:00 AM versus 10:30 AM for MCR: negative cluster left occipitally (interval 80–140 ms (*p* = 0.02)) and the P30 10:30 AM versus 1:00 PM for MCL: negative cluster right frontally (interval 20–35 ms (*p* = 0.02)). The TEP after repeating the TMS protocol a week later also closely resembles the TEP from a week earlier (see Fig. 4).

**Fig. 5:**
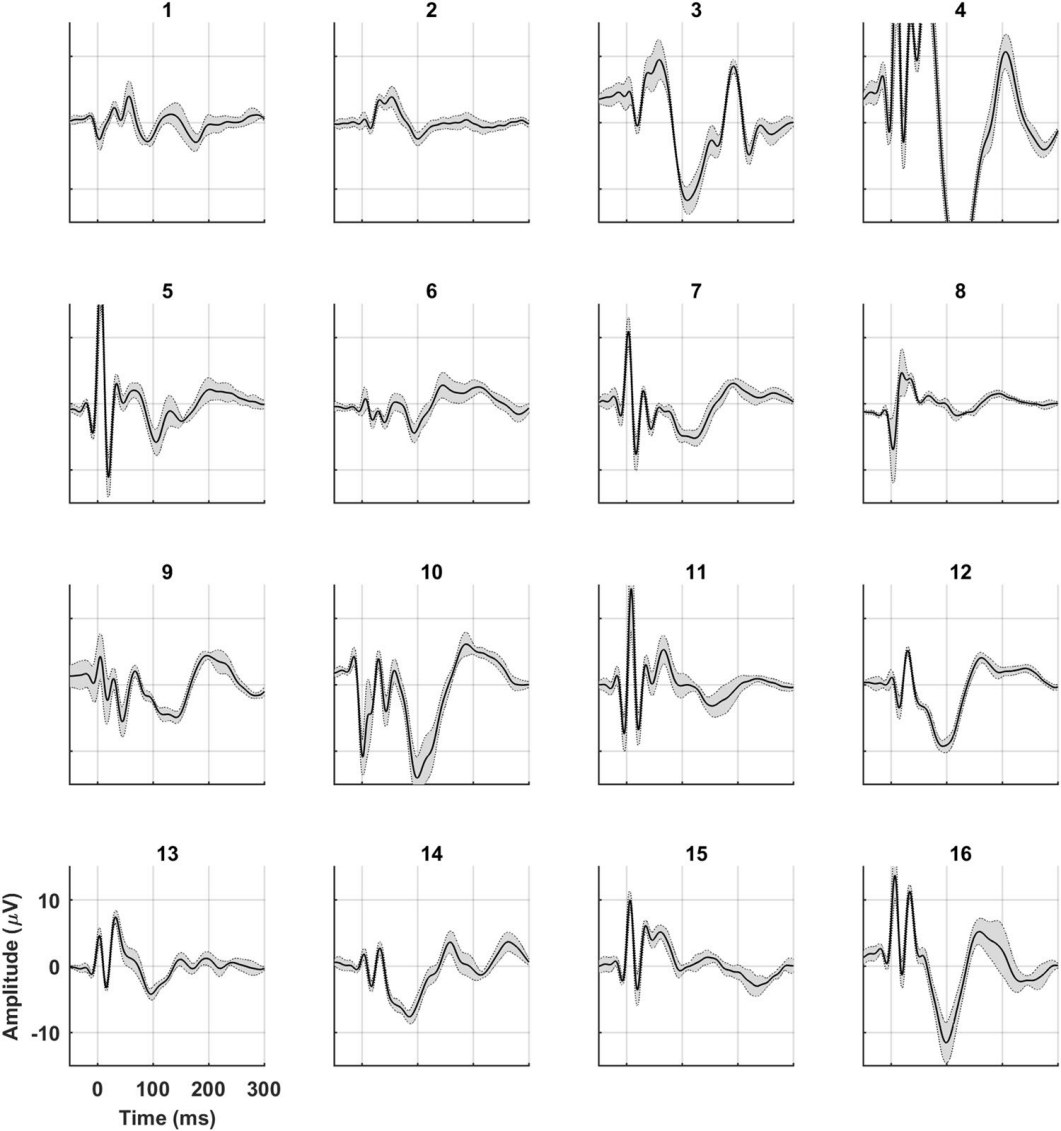
TEP on single subject level during the day. The mean TEP on electrode Cz during the day for all single subjects after stimulating the right motor cortex. The dotted lines represents the standard deviation. Numbers above the graphs indicate the subject number

We calculated the mean TEP with standard deviation over all subjects in the five sessions. In Fig. 2 the mean TEP with standard deviation at Cz is shown for left motor stimulation for session 1. The average standard deviation of the N100 component at Cz on a group level during the day was 4.53 µV (range 3.99–5.19 µV) for MCL and 6.16 µV (range 5.40–7.02 µV) for MCR. The mean TEP with SD over five sessions for each single subject is shown in Fig. 5. The average standard deviation of the N100 component at Cz on a single subject level during the day was 2.01 µV (range 0.40–4.27 µV) for MCL and 1.35 µV (range 0.27–3.11 µV) for MCR. The average standard deviation of the P30 component at Cz on a group level during the day was 5.45 µV (range 4.36–6.98 µV) for MCL and 4.42 µV (range 4.04–4.71 µV) for MCR. The average standard deviation of the P30 component at Cz on a single subject level during the day was 2.98 µV (range 0.58–8.48 µV) for MCL and 1.14 µV (range 0.36–2.73 µV) for MCR. Both for the N100 and the P30 the standard deviation on group level was significantly higher than the standard deviation on single subject level during the day (P30 MCL: *p* = 0.002; P30 MCR, N100 MCL, N100 MCR: all *p* < 0.001).

## Discussion

In this study we applied TMS–EEG five times during the day in healthy volunteers, and measured the RMT, MEP amplitude and the TEP. We found that both RMT and MEP amplitude do not change significantly during the day. The TEP remained largely constant, except for the N100 which was more pronounced at 3:30 PM compared to 10:30 AM. The inter-individual variation of the TEP within one session is significantly larger than the intra-individual variation during the day.

The absence of significant variation of the RMT between two sessions on 1 day has been reported before (Doeltgen and Ridding 2010; Lang et al. 2011; Tamm et al. 2009). Measurements at multiple time points in a 10 h period showed that the RMT stays constant (Koski et al. 2005), and this stability of the RMT during daytime is now confirmed by our results. Practically, this means that in healthy volunteers the RMT only has to be determined at the start of a TMS session, even when this TMS session takes very long or consists of multiple measurements within a day. In our data the MEP amplitude did not change over sessions, in line with a previous report measuring the MEP over a 24 h period (Strutton et al. 2003).

The TEP had a characteristic waveform in all subjects with recognizable components at 15–30–45–60–100–180 ms. This response was very constant during the day, with only the N100 after MCL stimulation showing a significant difference between the session at 10:30 AM and 3:30 PM on a group level. This cluster was located left occipitally, just as the two other N100 clusters that turned out to be non-significant after correction. Two earlier reports did not find a significant change of the first large component of the TEP (P30) during daytime (Huber et al. 2012; Ly et al. 2016). However, these authors did not analyze any other components. We evaluated the whole TEP waveform on all electrodes, and found a significant difference in a different component, the N100. All other components, including the P30, did not show significant differences. We only found a non-significant trend for the P30, with a larger amplitude at 1:00 PM compared to 10:30 AM, which is in line with previous findings of a non-significant increase of P30 amplitude during daytime (Huber et al. 2012; Ly et al. 2016). The TEP has shown to be reproducible after 1 week (Casarotto et al. 2010; Lioumis et al. 2009), similar to our observations in three subjects.

Other TMS measures were also reported as being constant during the day, such as short interval cortical inhibition (SICI) and intra cortical facilitation (ICF) (Doeltgen and Ridding 2010; Lang et al. 2011; Pfutze et al. 2007). Both SICI and ICF are GABA-A mediated responses (Hanajima et al. 1998; Inghilleri et al. 1996; Kujirai et al. 1993). On the contrary, long interval cortical inhibition (LICI) as well as the cortical silent period (CSP) decreased in length during three sessions at 8 AM, 2 PM and 8 PM (Lang et al. 2011), suggesting that the amount of inhibition decreases during the day. Earlier studies did not find a significant change in CSP during seven sessions between 8 AM and 8 PM (Koski et al. 2005) or between an evening and a morning session (Pfutze et al. 2007). LICI is a GABA-B mediated response (McDonnell et al. 2006; Pierantozzi et al. 2004; Werhahn et al. 1999), while CSP is thought to consist of a GABA-A mediated part and a GABA-B mediated part (Inghilleri et al. 1996; Kimiskidis et al. 2006; Siebner et al. 1998; Stetkarova and Kofler 2013). The N100 has also shown to be GABA-B mediated (Premoli et al. 2014), and in our results the N100 was larger in amplitude in the afternoon compared to the morning, suggesting an increase instead of decrease in inhibition. Taking these results together, it appears that GABA-A mediated TMS responses do not fluctuate during the day, while for the GABA-B mediated LICI and N100, and the partly GABA-B mediated CSP, inconsistent results are found.

Contradicting results are reported for the time-dependency of cortical excitability measured by long-interval cortical inhibition (LICI) in JME. Excitability was decreased in the afternoon compared to the morning in drug-naïve JME patients (Badawy et al. 2009), but no difference in excitability was reported in an earlier study (Pfutze et al. 2007). In addition, a difference in excitability between morning and afternoon could not be found in a group of focal epilepsy (mainly temporal lobe epilepsy) patients (Badawy et al. 2009). This inconsistency in TMS findings in healthy subjects and epilepsy patients may be explained by differences in methodology, for example the (lack of) control of different Zeitgebers or the heterogeneity of subjects with regard to morning type and evening type. True circadian or daytime variations may only be found using a constant routine methodology (Duffy and Dijk 2002). In any case, for a TMS readout to be of clinical use, the influence of normal, daily variations should be small compared to the influence of a disease or a medication.

We further show that intra-individual variation during the day of the N100 and P30 amplitude was significantly smaller compared to the inter-individual variation within a session. This is in line with a previous report describing a large inter-individual variation in TMS measures (Koski et al. 2005). Large differences between subjects have also been described in recent publications evaluating the TEP before and after different (anti-epileptic) drugs (Premoli et al. 2017, 2014).

Our study has a few limitations. Some subjects had a very high RMT at the start and therefore it was not possible to use a relative stimulation intensity of 110% during session 1. Because we compared the TEP between sessions, and not between subjects, and the TEP is also present at stimulation intensities below RMT (Komssi et al. 2004), this most likely had no effect on the results. We used the same stimulation intensity during all five sessions, even though there were small (non-significant) changes in RMT between sessions. MEP amplitude, directly dependent on RMT, has been related to the amplitude of the N15-P30 and N100 component (Mäki and Ilmoniemi 2010b; Paus et al. 2001) and to a late response around 300 ms (Fecchio et al. 2017). However, there were no significant differences in MEP amplitude between sessions. On the one hand the constant stimulation intensity may have influenced the TEP amplitude, since the relative stimulation intensity deviated from 110% in sessions 2–5. Still, this did not result in a difference in TEP waveform during the day, probably also because the relative stimulation intensity was sometimes above and sometimes below 110%. On the other hand, keeping the stimulation intensity constant ensured that the contribution of auditory and somatosensory evoked potentials and muscle activation artifacts was similar for all sessions and did not influence our findings.

We used PCA to reduce the TMS pulse artifact and the muscle artifact from our data. Although both are not designed for time-locked data such as evoked potentials, ICA (Hamidi et al. 2010; Iwahashi et al. 2008; Korhonen et al. 2011; Rogasch et al. 2014) and PCA (Hernandez-Pavon et al. 2012; Mäki and Ilmoniemi 2010a; Rogasch et al. 2017) have been successfully used for removing artifacts from TMS–EEG. Also other techniques (Casula et al. 2017; Litvak et al. 2007; Morbidi et al. 2007) have been applied. All methods result in a reduction of TMS-related artifacts and thereby enable TEP analysis. Although we have shown that PCA is an effective method to reduce the TMS pulse artifact and the muscle artifact simultaneously (ter Braack et al. 2013), the method can possibly be further optimized. Indeed, some residual artifacts were still present, causing minor filtering effects around the time of the TMS pulse as can be seen in Figs. 2, 3, 4 and 5. We now removed the first four components in all subjects, but it may be better to decide on the number of components to be removed on a single subject level. When increasingly more PCA components are removed, the TEP components that have the same direction as the artifact also decrease in amplitude (Mäki and Ilmoniemi 2010a; ter Braack et al. 2013). Earlier research on the same dataset showed that our PCA approach reduces the TEP component around 100 ms (ter Braack et al. 2013). This is the time-frame where we found significant differences for a parieto-occipital cluster. Therefore, it has to be considered that the PCA filtering could have suppressed the TEP at areas where the TMS-artifact was more expressed, resulting in the appearance of a null-finding over e.g. motor areas. A larger reduction of the TEP over the stimulation area was also reported by other authors using PCA filtering (Mäki and Ilmoniemi 2010a). Despite of these drawbacks (Mutanen et al. 2016), PCA is still suggested as an effective method to reduce the large muscle artifacts before evaluating the TEP more closely (Rogasch et al. 2017). It is likely that a combination of different methods is needed to remove all artifacts while not affecting the TEP (Atluri et al. 2016; Rogasch et al. 2017; Wu et al. 2018).

In conclusion, we show that the RMT, MEP amplitude and TEP in healthy subjects are highly reproducible during daytime. No significant differences were found for both RMT and MEP amplitude, while only the N100 amplitude after left motor cortex stimulation was significantly larger at 3:30 PM compared to 10:30 AM. This implies that results from different sessions can be compared even though they are obtained at a different time of day. Large inter-individual differences still may cause difficulties in establishing normal ranges for TMS measures, which is important for developing clinical applications.
